# The Relevance of Marine Chemical Ecology to Plankton and Ecosystem Function: An Emerging Field

**DOI:** 10.3390/md9091625

**Published:** 2011-09-22

**Authors:** Adrianna Ianora, Matthew G. Bentley, Gary S. Caldwell, Raffaella Casotti, Allan D. Cembella, Jonna Engström-Öst, Claudia Halsband, Eva Sonnenschein, Catherine Legrand, Carole A. Llewellyn, Aistë Paldavičienë, Renata Pilkaityte, Georg Pohnert, Arturas Razinkovas, Giovanna Romano, Urban Tillmann, Diana Vaiciute

**Affiliations:** 1 Stazione Zoologica Anton Dohrn, Villa Comunale Napoli 80121, Italy; E-Mails: raffa@szn.it (R.C.); romano@szn.it (G.R.); 2 School of Marine Science and Technology, Newcastle University, Ridley Building, Claremont Road, Newcastle upon Tyne, Tyne and Wear, England NE1 7RU, UK; E-Mails: m.g.bentley@ncl.ac.uk (M.G.B.); gary.caldwell@ncl.ac.uk (G.S.C.); 3 Alfred Wegener Institute for Polar Research, Am Handelshafen 12, Bremerhaven 27570, Germany; E-Mails: Allan.Cembella@awi.de (A.D.C.); Urban.Tillmann@awi.de (U.T.); 4 ARONIA Research and Development Institute, Novia University of Applied Sciences & Åbo Akademi University, Raseborgsvägen 9, Ekenäs FI-10600, Finland; E-Mail: jonna.engstrom-ost@nova.fi; 5 Plymouth Marine Laboratory, Prospect Place, The Hoe, Plymouth PL1 3DH, UK; E-Mails: clau1@pml.ac.uk (C.H.); CALL@pml.ac.uk (C.A.L.); 6 International Max Planck Research School of Marine Microbiology, Celsiusstrasse 1, Bremen 28359, Germany; E-Mail: eva.c.sonnenschein@gmail.com; 7 School of Natural Sciences, Linnaeus University, Kalmar 352 52, Sweden; E-Mail: catherine.legrand@lnu.se; 8 Coastal Research and Planning Institute, Klaipeda University, Manto 84, Klaipeda LT-5802, Lithuania; E-Mails: aiste@corpi.ku.lt (A.P.); renata@corpi.ku.lt (R.P.); art@corpi.ku.lt (A.R.); diana@corpi.ku.lt (D.V.); 9 Institute for Inorganic and Analytical Chemistry, Friedrich Schiller University Jena, Jena D-07743, Germany; E-Mail: georg.pohnert@uni-jena.de

**Keywords:** allelopathy, biotoxins, signal molecule, teratogen, toxic algae

## Abstract

Marine chemical ecology comprises the study of the production and interaction of bioactive molecules affecting organism behavior and function. Here we focus on bioactive compounds and interactions associated with phytoplankton, particularly bloom-forming diatoms, prymnesiophytes and dinoflagellates. Planktonic bioactive metabolites are structurally and functionally diverse and some may have multiple simultaneous functions including roles in chemical defense (antipredator, allelopathic and antibacterial compounds), and/or cell-to-cell signaling (e.g., polyunsaturated aldehydes (PUAs) of diatoms). Among inducible chemical defenses in response to grazing, there is high species-specific variability in the effects on grazers, ranging from severe physical incapacitation and/or death to no apparent physiological response, depending on predator susceptibility and detoxification capability. Most bioactive compounds are present in very low concentrations, in both the producing organism and the surrounding aqueous medium. Furthermore, bioactivity may be subject to synergistic interactions with other natural and anthropogenic environmental toxicants. Most, if not all phycotoxins are classic secondary metabolites, but many other bioactive metabolites are simple molecules derived from primary metabolism (e.g., PUAs in diatoms, dimethylsulfoniopropionate (DMSP) in prymnesiophytes). Producing cells do not seem to suffer physiological impact due to their synthesis. Functional genome sequence data and gene expression analysis will provide insights into regulatory and metabolic pathways in producer organisms, as well as identification of mechanisms of action in target organisms. Understanding chemical ecological responses to environmental triggers and chemically-mediated species interactions will help define crucial chemical and molecular processes that help maintain biodiversity and ecosystem functionality.

## 1. Introduction

Chemical ecology as an integrative science has been instrumental in understanding important mechanisms underlying ecosystem functioning. There is growing recognition of the central role that chemical ecology plays in maintaining the structure, function and balance of plankton ecosystems. Many key life processes including: food source identification and selectivity; prey location and capture; mate recognition and location; chemical defense; behavior; and population synchronisation are mediated by chemical interactions. A scenario whereby such chemical stimuli were removed would result in a catastrophic cascade of disruption to inter- and intra-specific interactions at individual, population and community levels.

Empirical research and conceptual insights over the past decade have shown that microbial and food web interactions in the plankton are far more complex than previously reported [1–4]. In light of this, there is a requirement to re-evaluate how planktonic ecosystems function. New approaches to interpret mechanisms that cannot be explained by classical food web and nutrient limitation models, which have often focused on biomass transfer driven by “bottom up” processes, are urgently needed. For example, toxic algal blooms are manifestations of how chemical activity of aquatic organisms can directly affect environmental and human health due to production and accumulation of nuisance or toxic metabolites. Yet these phenomena are typically not amenable to plankton dynamic modeling appropriate for defining bloom characteristics and associated trophic interactions.

In contrast to the relatively well-studied chemical interactions between benthic plant metabolites and herbivores [5], the effects of microbial secondary metabolites on planktonic herbivores are not well known and remain under-investigated. As is the case in benthic ecosystems, in the pelagic realm, potential prey must escape from hunters, deter predation, or tolerate herbivory. In this essay we focus on some of the better studied chemically-mediated interactions, and primarily consider cases for which the specific compounds have been identified and are available in pure or semi-purified form for manipulative experiments. We also compare theories of plant-herbivore interaction for the plankton with those developed for terrestrial communities. Finally, we address some of the challenges and opportunities for future research in plankton chemical ecology, incorporating recent advances in genome sequencing and gene expression.

## 2. Chemical Cues and Phytoplankton-Zooplankton Interactions

Although identification of bioactive metabolites in an ecological context has been lagging in the plankton, the few compounds that have been isolated and characterized have been drivers of intensive research efforts, yielding new concepts and advances in the field. Verifying hypotheses with pure compounds has the power to generate paradigm shifts. This has been demonstrated, for example, for polyunsaturated aldehydes (PUAs) and other products that, like PUAs, derive from the oxidation of fatty acids (collectively termed oxylipins). PUAs were first discovered in marine [6] and freshwater [7] diatoms a little over a decade ago. Other oxylipins such as hydroxyacids and epoxyalcohols have been discovered even more recently [8–10]. To date, fourteen oxylipin derivatives have been structurally identified in marine planktonic diatoms [11]. These metabolites impact food webs by interfering with the reproductive success of herbivores (reviewed by [11,12–15] thereby introducing a new perspective into phytoplankton-zooplankton interactions.

The PUA decadienal is the best studied metabolite of this group and thus has become a model aldehyde for experimental studies on the effects of oxylipins on marine organisms. Numerous functions have been proposed for this highly reactive molecule, such as: grazer defense [6,16]; allelopathy [17]; cell to cell signaling [18]; antibacterial activity [19,20]; and bloom termination initiator [9,21,22]. These functions are summarized in Table 1, which presents a synopsis of the effects of PUAs and other microalgal metabolites on predators, competitors and pathogens in the plankton.

The PUAs are of great interest to plankton ecologists because of their teratogenic activity, which leads to structural malformations in the offspring of organisms exposed to them during gestation [13,16]. The structural malformations that can occur include: fetal growth retardation, embryo and fetal mortality, and functional impairment due to malformed limbs or organs. Although the effects of such toxins are less catastrophic than those inducing acute poisoning and death, such as the dinoflagellate neurotoxins, they are nonetheless insidious and can reduce the predator’s overall fitness. Bloom dynamics are positively impacted through induced abortions, birth defects and reduced larval survivorship of potential predators thereby releasing diatom blooms from grazing pressure that would otherwise have caused them to crash. Toxic PUAs are also of great interest because of their possible function as a diffusible bloom-termination signal that triggers active cell death in diatoms [18] and because they act as allelopaths affecting growth and physiological performance of diatoms and other phytoplankton [17].

Induced chemical defenses in response to grazing have also been shown for the bloom-forming marine coccolithophorid, *Emiliana huxleyi*, which produces dimethylsulfoniopropionate (DMSP) primarily as an osmolyte. Protistan and zooplankton grazers feeding on *E. huxleyi* induce cleavage of DMSP by lyase enzymes to form the gas dimethylsulfide (DMS) and acrylate [34]. DMSP appears to act as a chemical cue or indicator of inferior prey, rather than as a toxin. Fredrickson and Strom [35] have shown that adding DMSP (20 mM) to laboratory cultures of two ciliates (*Strombidinopsis acuminatum* and *Favella* sp.) and one dinoflagellate (*Noctiluca scintillans*) causes a 28–75% decrease in feeding rates and that these decreases were concentration-dependent with 20 nM as the lower threshold for an effect. Partial but not complete recovery of grazing rates occurred during long-term (24 h) exposure to dissolved DMSP, as long as concentrations remained above 12 mM.

There are other studies showing that DMSP-producing species of phytoplankton do not deter grazers suggesting that some predators are less sensitive to or more capable of detoxifying this metabolite. *Emiliania huxleyi* contains high intracellular levels of DMSP yet some strains of *E. huxleyi* are a suitable food source for protist grazers [36–38]. Copepods also consume *E. huxleyi*, though not as a preferred food source [37]. Like *E. huxleyi*, the prymnesiophyte *Phaeocystis* is a suitable food source for some protist grazers even if it is a strong DMSP producer [39]. However, studies of copepod feeding upon *Phaeocystis* generally give varied results, many of which related to sizes of colonies, differences between copepod species and experimental techniques. Turner *et al.* [40] concluded that even though copepods may feed well upon *Phaeocystis*, resulting poor fecundity on this diet may inhibit copepod population increases during blooms, thereby contributing to the perpetuation of blooms.

DMS derived from the breakdown of DMSP also shows biological activity. A wide range of organisms seem to use DMS as a cue for food finding [41–43]. It is possible that DMS odor plumes produced during zooplankton grazing are used by predators to detect, locate and capture their prey. Plumes of DMS trigger a tail-flapping response in the copepod *Temora longicornis* which results in altered flow patterns and probably assists copepods in locating food [28]. The identification of the involved signal is important for general considerations about food finding by copepods. Since grazers are involved in the cell disintegration that triggers DMS production, this process could attract herbivores to patches with high food concentrations and in turn assist predation on actively feeding herbivores [43]. Carnivores can also benefit from the production of these infochemicals (information-conveying chemicals) which they sense and use to detect herbivores thereby reducing the grazing pressure exerted by the herbivores on the plants [41]. Very little is known on such tritrophic (involving three trophic (feeding) levels) interactions in the plankton.

Oxylipins and DMSP are produced by a variety of phytoplankton species and are widely distributed in the plankton [11,44]. The PUAs are found in both planktonic [23], and benthic [10] diatoms, and have also been reported in the marine prymnesiophyte *Phaeocystis pouchetii* [45] and in a number of freshwater diatoms and chrysophytes [7,14]. Other oxylipins (hydroxyacids and epoxyalcohols) are found in both PUA-producing and non-producing diatoms [8–10]. In comparison, DMSP is particularly enriched in marine flagellates, including several prymnesiophytes (*Chrysochromulina*, *Phaeocystis* and *Emiliania*) and dinoflagellates (*Alexandrium*, *Amphidinium*, *Gonyaulax* and *Gymnodinium*) [44]. Despite their abundance and obvious presence in the plankton we still know little about what regulates production of these metabolites and their direct delivery to the interacting organisms.

In addition to these classes of compounds, certain paralytic shellfish toxins (PST) contained especially in dinoflagellates, prymnesiophyes, raphidophytes and other phytoplankton, induce strong physiological responses in copepods after <24 h of feeding on these cells. Effects range from elevated heart rates, regurgitation, loss of motor control and twitching of the mouthparts [46], decreased feeding [47], decreased fecundity [48], delayed development [49] and direct mortality [50]. Furthermore, the copepods *Calanus finmarchicus* [51], *Eurytemora herdmanii* and *Acartia tonsa* [52] have been shown to avoid feeding on PST-containing prey when offered together with a nontoxic food option. Based on these findings and the negative effects that PST containing phytoplankton have on some grazers, PSTs have been suggested to act as a protection against grazers [52], as also suggested by studies showing that their concentration in the cell increases following contact with herbivore-specific chemicals [53]. This has been shown for the marine copepod *A. tonsa* whereby waterborne chemicals produced by this copepod caused an increase in PST production in the dinoflagellate *Alexandrium minutum* which produces saxitoxins [31]. *A. minutum* contained up to 2.5 times more toxins than controls and was more resistant to further copepod grazing. Further investigations [31,53,54] showed that when *A. minutum* was grown under nitrate-rich conditions, but not in low nitrate treatments, the presence of waterborne cues from grazers resulted in significantly increased cell-specific toxin content, implying that the magnitude of grazer-induced PST production was directly proportional to the degree of nitrogen availability. This response was also grazer-specific, with some species of copepods inducing a higher production of toxins (up to 20-fold increase in the presence of the copepod *Centropages typicus*) than others, which induced little or no change at all (*Pseudocalanus* sp.) [53]. Exposure to grazer-specific cues has also been observed to induce behavioral changes in *A. minutum* with long chains of cells separating to form smaller chains or even single cells. These changes were matched by reduced swimming velocities of the single cells relative to longer chains [55]. The ability of *A. minutum* to sense and respond to the presence of grazers by increased PST production, and the associated negative effect on grazers, is strong evidence that these compounds are defensive metabolites the purpose of which is not necessarily to intoxicate and kill the predator but to discourage further consumption.

Using a functional genomics approach that involves the regulation of serine/threonine kinase signaling pathways [56] provides further evidence for the defensive role of PST toxins and the ability of phytoplankton to recognize a specific copepod as a threat. When *Alexandrium tamarense* was exposed to three copepod species (*Calanus helgolandicus*, *Acartia clausii*, and *Oithona similis*) and their corresponding waterborne cues, not all of the tested copepod species triggered an increase in toxin production (e.g., *O. similis* induced no response in gene expression levels) indicating that the species-specific alarm cues were not activated when non-threatening copepods were present. Toxin production was correlated with both the presence of and cues from copepods that had high grazing impact on *A. tamarense* (*C. helgolandicus*) which triggered gene regulation for a larger range of genes and not only the genes directly involved in defense. Providing further evidence of activity at the gene level, Innes *et al.* [57] using a microarray approach observed that 14 genes from *A. minutum* were activated in response to grazer cues.

Another toxin producing dinoflagellate *Karenia brevis* produces brevetoxins, polyketide neurotoxins with acute toxic activity in waterborne or aerosol form against vertebrates [58]. However, according to Prince *et al.* [59], negative effects of *K. brevis* on copepod egg production and survivability are not due to a chemical deterrent, but likely caused by the nutritional inadequacy of *K. brevis* as a food source. In contrast, Kubanek *et al.* [60] showed that grazing deterrence of rotifers by *K. brevis* was chemically-mediated. The nature of the chemical defense was not identified however it was determined that brevetoxins were not the deterrents.

Blooms of the haptophyte *Prymnesium parvum* are well known nuisances in brackish waters around the world that are usually accompanied by massive fish kills due to the production of prymnesins which exhibit potent cytotoxic, hemolytic, neurotoxic and ichthyotoxic effects. These secondary metabolites are especially damaging to gill-breathing organisms and they are believed to interact directly with plasma membranes, compromising integrity by permitting ion leakage. Several factors appear to function in the activation and potency of prymnesins including salinity, pH, ion availability, and growth phase [61]. Prymnesins may function as defense compounds to prevent herbivory and some investigations suggest that they have allelopathic roles, although evidence remains equivocal due to lack of access to the purified compounds. Exposure to *P. parvum* can cause inactivity in the copepods *Eurytemora affinis* and *Acartia bifflosa*, without the copepods actually consuming the toxic alga, resulting in reduced copepod reproductive success [62]. In further experiments [63] found that cell-free filtrates of *P. parvum* also negatively impacted copepod survivorship.

Another class of feeding deterrents in the plankton is the apo-fucoxanthinoids produced by the diatoms *Phaeodactylum tricornutum* and *Thalassiosira pseudonana* [29]. Feeding deterrent responses in the copepod *Tigriopus californicus* were observed at concentrations which were about 1000 times lower than the total apo-fucoxanthinoid concentration in *P. tricornutum* [30]. Thus these compounds are present in concentrations which may have ecological significance in the control of bloom formation and grazing even if *P. tricornutum* and *T. pseudonana* are not major blooming species.

Apart from these unrelated classes of compounds that have been identified in the plankton until now, many more novel roles for bioactive metabolites are postulated for the plankton (reviewed by [15,64,65]). Most of these metabolites are present in very low concentrations, both in the producing organism and in the surrounding aqueous medium. This poses a severe challenge for their detection, structural elucidation and quantification. Furthermore, their bioactivity may be subject to synergistic interactions with other natural and anthropogenic environmental toxicants that can cause harmful effects, such as changes in the immune system, behavioral alterations, and impaired reproduction [66]. Chemical stressors including ocean acidification and increased temperatures can also alter the production and degradation of these products [67]. This is particularly important in pristine ecosystems such as polar oceans, where environmental change is expected to occur earlier due to their higher vulnerability and have the largest impact, yet information on trophic and chemical interactions is extremely scarce.

There is high species-specific variability in the effects of toxins on grazers, with effects ranging from severe physical incapacitation and death in some species to no apparent physiological effects in others [50]. This variability may depend on the capability of some predators to detoxify these compounds or indeed whether there is a shared evolutionary/biogeographic history such as described by Kubanek *et al.* [60] for *K. brevis* and rotifers. Rotifers with a shared history had higher tolerance to *K. brevis* than rotifers from locations with no *K. brevis* blooms. There is increasing evidence for the importance of key detoxification enzymes such as P450s, glutathione S-transferases and ATP Binding Cassette (ABC) transporters , with broad substrate specificities that may convert secondary metabolites into highly water-soluble, less toxic products and eliminate them from the cell and body [68]. At the same time, each herbivore species is likely to show unique features in detoxification/transport mechanisms as is the case for detoxification by insect. Colin and Dam [69,70] have shown that when two geographically distant populations of the copepod *Acartia hudsonica* were reared on the toxic dinoflagellate *Alexandrium fundyense*, the one that had not experienced recurrent blooms of the toxic algae had lower somatic growth, size at maturity, egg production, and survival, compared to the other population that showed no effects on these life history parameters. Some copepod species also seem capable of concentrating toxins in their body tissues [71,72]. This creates the opportunity for epimerization (bio-transformations) that may yield the formation of more potent analogues of these toxins, as frequently occurs in mollusks [73]. Although there is no evidence that copepods are capable of long term sequestration of such toxins, these predators provide not only a link for toxin flux in pelagic food webs but they may also act as a sink for toxins by metabolizing and removing them from the environment. Provided that they are not incapacitated by the toxin, in some cases the accumulated toxin may even serve as a transient defense against more sensitive predators.

During the last two decades, bioassays have progressed from those that detected bioactivity without much concern for ecological relevance, to ecologically relevant tests to investigate possible functions of secondary metabolites in the phytoplankton. The problem is that often the natural concentrations of a compound, and, therefore the concentrations that should be tested, are not known. Most phytoplankton secondary metabolites have been isolated and identified by natural products chemists looking for unusual secondary metabolites. Rarely have chemical studies provided information on the yield of these compounds after extraction. Hence it is difficult for ecologists conducting bioassay experiments to know the natural concentrations of these metabolites to be tested. To further complicate matters, there are geographical variations in the concentration of secondary metabolites and within-region variation in some cases.

Notwithstanding the above obstacles, much progress has been made in recent years in designing ecologically relevant bioassays with natural concentrations of a compound in many feeding trials (e.g., [31]). A recent study has explored the possibility of using liposomes as a delivery system for copepods [74]. Giant liposomes were prepared and characterized in the same size range of food ingested by copepods (mean diameter = 7 μm) and then encapsulated with decadienal in order to investigate the effect of PUAs on the reproductive biology of the copepods *Temora stylifera* and *Calanus helgolandicus*. After 10 days of feeding, liposomes reduced egg hatching success and female survival with a concomitant appearance of apoptosis in both copepod embryos and female tissues. Concentrations of decadienal inducing blockage of cell divisions were one order of magnitude lower than those used in classical feeding experiments (e.g., [16]) demonstrating that liposomes were a useful tool to quantitatively analyze the impact of toxins on copepods. This type of tool for the delivery of toxins and drugs in feeding trials has considerable potential as a means of delivering a known quantity of toxin under semi-natural conditions [75]. The further development of liposome and similar bio-encapsulation technologies for application in chemical ecology should, where feasible, seek to counterbalance the effects of the test molecules by co-encapsulation with nutritionally attractive factors, e.g., amino and fatty acids. There is a school of thought that suggests that the efficacy of consuming a diet containing deterrent (negative) compounds can be eclipsed by the nutrients gained from the diet. This creates an interesting and for phytoplankton a potentially dynamic relationship between deterrent efficiency and intrinsic nutritional value. If reliable and reproducible co-encapsulation can be demonstrated it would pave the way for highly controlled feeding trials that could replicate algae cellular conditions as occurs throughout the course of a bloom and under variable environmental conditions.

## 3. Chemical Identification of Allelochemicals in Phytoplankton

Several bloom and/or PST-producing species produce allelopathic compounds to outcompete or at least co-exist with other species by inhibiting their growth, photosynthesis, or causing death [76]. In most cases, the chemical nature of the bioactive compounds remains unknown [52] and preliminary characterization is only available for a few PST-producing species [64,33,77]. Allelochemicals produced by the toxigenic dinoflagellate *Alexandrium* are clearly distinct from well-known neurotoxins (*i.e.*, paralytic shellfish toxins, [78] or spirolides [79] produced among many strains within this genus. In *A. tamarense* these compounds are presumably large molecules (>5 kD), stable over broad temperature and pH ranges, and refractory to bacterial degradation [33]. *Alexandrium* allelochemicals have lytic activity targeting both competitors and grazers, underlying the success of this species during blooms [80].

Certain strains of the dinoflagellate *Karlodinium* produce karlotoxins, a group of ichthyotoxins also causing membrane permeabilization in other protists [24]. Karlotoxins are linear polyketides with lytic properties similar to those of amphidinols produced by the dinoflagellate, *Amphidinium* [81]. In the raphidophyte *Heterosigma akashiwo*, a still uncharacterized polysaccharide-protein complex has been shown to be responsible for the growth inhibitory effect on the competitor diatom *Skeletonema costatum* [82].

The dinoflagellate *Karenia brevis* has also been reported to negatively affect the growth rate of most sympatric and temporally co-occurring competitors, while enhancing the growth for some other species [83]. These authors suggested that this species may have evolved resistance against the allelochemicals of their competitors. Prince *et al.* [84] also found that exposure to waterborne compounds from *K. brevis* resulted in growth inhibition or death for four of five co-occurring species tested, whereas compounds exuded by *K. brevis* cultures suppressed three of these same competitors. *K. brevis* exudates lowered photosynthetic efficiency and damaged cell membranes of competing phytoplankton, but had no effect on the esterase activity of competitors, nor did they limit competitor access to iron. Studies by Prince *et al.* [85] and Poulson *et al.* [86] indicate that allelopathy due to *K. brevis* may not be as straightforward as a number of laboratory-based studies would suggest. The production of allelopathic compounds varied with biotic interactions and some diatom competitors appeared capable of circumventing the allelopathic impacts, indeed in some instances the growth of the diatoms was increased. The absolute chemical identity of these compounds remains unknown however preliminary characterization indicates they are between 500 and 1000 Da and possess aromatic functional groups [77].

The prymnesiophyte *Prymnesium parvum* produces prymnesins with lytic activity against protists [87,88]. The number of different substances responsible for these toxic effects and the mode of action are currently unknown. Although two putative polyketides; prymnesin 1 and prymnesin 2 with toxic activity were isolated and structurally characterized from *P. parvum* [89], the extent to which they can account for toxic and allelochemical interactions of this species remains unclear. Other bioactive compounds of *Prymnesium* have also been characterized, including: glycolipids; galactolipids; proteolipids; and lipid-carbohydrate complexes [61]. Recent reports confirmed the discrepancies between haemolytic and ichthyotoxic activities of different fractions of *Prymnesium* exudates [90], thereby demonstrating the diversity and complexity of these secondary metabolites.

Finally diatom PUAs that are reported to induce reproductive failure in predators (see previous section) also seem to act as allelochemicals by suppressing the growth of other phytoplankton [91]. Interestingly, one of the target species, the PUA-producing diatom *S. marinoi* was less affected by these compounds than other target species, suggesting that this diatom is partially resistant to the compounds it produces. Decadienal has been shown to trigger the generation of nitric oxide (NO) which results in cell death [18]. Pre-treatment of cells with sublethal doses of decadienal induces resistance to subsequent lethal doses demonstrating the existence of a sophisticated stress surveillance system in diatoms which allows diatom cells to sense local PUA concentrations and integrate this information in a temporal context. According to these authors, when stress conditions are aggravated during a bloom, PUA concentrations could exceed a certain threshold and act as a diffusible bloom-termination signal triggering population-level cell death.

Most studies testing allelopathy in plankton have used cell-free filtrates or cell extracts of unknown composition (e.g., [79]), however the structural identification of allelochemicals is needed to explore in depth the nature of allelopathic interactions among plankton, and whether allelopathic effects are general or species-specific. There is increasing awareness of the importance of allelochemistry in marine ecosystem functioning and biodiversity (reviewed by [92]). Apart from the chemical identification of the molecules involved, future challenges include the identification of biosynthetic pathways in defense mechanisms in phytoplankton and the understanding of the transcriptional changes and signal transduction mechanisms occurring during biotic interactions. In this context, genomic approaches are very promising.

## 4. Can Concepts of Terrestrial Chemical Ecology Be Directly Applied to the Plankton?

**Figure 1:**
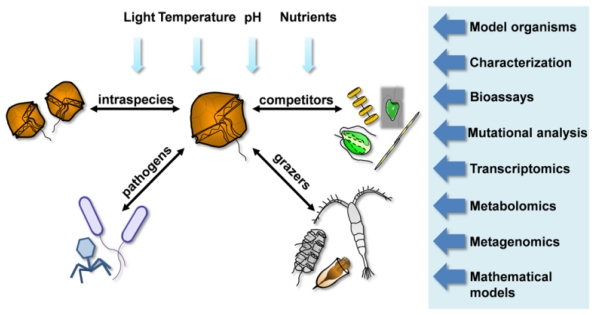
Pathways of chemical interactions between a species and its environment demonstrated for the dinoflagellate *Alexandrium tamarense* and methods that are necessary to address this topic. Interactions include: intra- and interspecific competition potentially involving allelopathy particularly within a bloom situation, resistance and resilience to infection, and capacity to deter grazing. All chemical interaction pathways must be considered within the framework of changing abiotic conditions.

Plants dominate terrestrial ecosystems yet the importance of plant biomass for the global ocean is perhaps less obvious. Due to the rapid turnover of phytoplankton biomass the ocean contains less than one per cent of plant biomass as standing stock. Despite this, in excess of 50% of global photosynthesis occurs in the oceans. Species are not evenly distributed spatially and fluctuate widely in abundance and diversity from year to year. In contrast to terrestrial ecosystems, species-specific interactions among planktonic organisms have not received much attention. Indeed, only a few examples of such interactions are reported in the literature (reviewed by [42,64]). Since a significant proportion of planktonic organisms are eventually eaten (either within the plankton system or as phytodetritus following sedimentation), one can expect a strong selection for mechanisms that reduce mortality within populations of a given species. Defense mechanisms can range from mechanical and structural [93] through avoidance behavior to various types of chemical warfare. The former two strategies have been well investigated and the mechanisms are explained at the empirical level. In contrast, given the paucity of information on chemical interactions in the plankton, more dedicated studies of the planktonic arms race [94] from a chemical ecological perspective are likely to reveal many types of previously unsuspected defense strategies involving chemical compounds (Figure 1).

One of the key arguments against the widespread existence of chemical ecological mechanisms (e.g., for defense) in the plankton is related to the assumed high metabolic cost [95] of biosynthesis and release of such elaborate bioactive compounds to the aqueous medium, with consequent high dilution effects [44]. It would appear that a high metabolic cost is unavoidable for phytoplankton. The higher abundance and diversity of membrane receptors in unicellular compared with multicellular organisms (*i.e.*, tissue forming) mean that lack of contact interactions and of a developed immunology must be compensated by a diverse chemical communication. Nevertheless, this “high cost of production”, generally referring to complex secondary metabolites, might be challenged for the plankton. Many of the active metabolites are simple molecules derived from primary metabolism (e.g., PUAs in diatoms, DMSP in prymnesiophytes). Furthermore, even plankton producing extremely elaborate metabolites do not seem to suffer retarded growth or other reductions in physiological processes [9]. Costs could be low and may be compensated for by the benefit of reduced predation risk [5]. An alternative explanation would be that some compounds, e.g., paralytic shellfish toxins, are retained within the cell allowing grazers to recognize and avoid high-toxin cells by association, thereby precluding the need to release and reconstitute defensive compounds.

The circumstances under which an organism invests in defense structures or metabolites may also differ for the plankton. Several explanatory models have been proposed for the production of defense metabolites in higher plants (e.g., the optimum defense and the plant apparency models). However, most of these models developed for multicellular organisms are not easy to apply to unicellular algae. Microalgae have no capacity for sacrificial tissues as required by the optimal defense hypothesis, and whereas microalgae may fall within the category of unapparent plants (*i.e.*, short lived plants that may invest in a modest chemical defense) their fit to the plant apparency model is weak. Macroalgae may however be more suited to these models. The models predict that younger individuals and younger parts of the organism should have higher levels of defense as these are under higher risk of being preyed upon as they are usually more nutritious. Similarly, this does not fit with microalgae chemical defense, for example PUA production is considerably enhanced with culture age and is often undetected during early stage growth.

In the plankton, a plausible explanatory model for the production of secondary metabolites used for defense is the carbon/nutrient balance hypothesis developed for boreal plants by Bryant *et al.* [96]. According to this model, most toxigenic algae produce their toxins mainly under conditions where carbon is in excess and other nutrients are limiting (reviewed by [97]). For example, the hemolytic activity of the prymnesiophytes *Prymnesium* and *Chrysochromulina* increases both under phosphorus and nitrogen limitation [98,99] and the production of PUAs also increases in nutrient-stressed diatoms [91]. Grazers feeding at the end of a bloom, when resources become limited, are therefore more likely to be affected than early-bloom grazers. Moreover, indirect effects of toxins should also be considered. For example, domoic acid is suggested to also regulate the availability of micronutrients such as copper and iron, by acting as a strong metal complexing organic ligand [100]. This is likely to occur in competition with siderophore production by bacteria contributing to complex microbial chemical interactions.

Similar to the carbon/nutrient balance hypothesis the growth rate hypothesis or resource availability hypothesis [101] may also be applied to phytoplankton under certain circumstances. This model relates to the growth rate of the organism which is inherently linked with resource availability. The model predicts that as resources become limiting and growth rate declines then investment in defense becomes metabolically affordable. This model may for instance be more appropriate for resource poor, *i.e.*, oligotrophic waters as opposed to coastal systems.

A further model worthy of consideration is the growth-differentiation balance hypothesis [102] which argues that defense is a function of growth-related and differentiation-related processes. Defenses are only produced by plants with an excess of available energy with the extent of the defense governed by a resource availability gradient. The differentiation function is perhaps less easy to argue for microalgae. However in respect of PUA production it may be posited that enhanced defense in later stage growth may coincide with a switch from the production of vegetative cells to the production of sexual cells or cysts, *i.e.*, a differentiation of cell function.

Mathematical models developed for chemical signaling in terrestrial ecosytems are usually based on drivers such as: variability, selection, population dynamics and genetics, but such models cannot be directly applied to pelagic ecosystems. This is primarily because of physical constraints within seawater such as: high viscosity, low diffusivity and mesoscale advective terms [44]; and small-scale turbulence that determine the sphere of influence and diffusivity of molecules [103]. Short-range chemical interactions and limited dispersion allow for coexistence of competing planktonic species that could not coexist in a well-mixed environment [104]. Among the different chemical interaction models, it is mostly those for allelopathic interactions that have been transposed from terrestrial to the planktonic component of aquatic systems [105]. Recent progress in understanding chemical interactions in the plankton has promoted the use of these models. However, the models are often based on a limited number of known parameters available in the ecological literature, with few species considered (e.g., [106]), and unknown nature/action of the allelochemicals. On the other hand, a few models have integrated field observations [107] and considered spatio-temporal variations instead of the traditional diffusion-reaction models [108]. Further, it may be prudent to look beyond terrestrial and multicellular plant-based models and focus more on microbial systems which share common features of cell and organism complexity, modes of growth and reproduction and, for many microbes, a similar aqueous-based habitat [109–113]. Development of chemical ecology models incorporating physical, temporal and spatial scale parameters should be a priority to better understand plankton interactions from the subcellular to population level and from the nanosecond to year temporal scales.

## 5. New Toolboxes to Study Chemical Interactions

The application of “omics” technologies (genomics, transcriptomics, proteomics, metabolomics, *etc*.) to studies of biosynthesis and regulation of bioactive secondary metabolites, and the concomitant effects on plankton population dynamics is an emerging but rapidly advancing field. Complete genome sequences have now been reported for a number of phytoplankton species [114] including the two diatoms, *Thalassiosira pseudonana* [115] and *Phaeodactylum tricornutum* [116] but others will soon be available for representative species of major groups (for example, heterotrophic and photosynthetic bacteria, prasinophytes, diatoms, *Emiliania huxleyi*, *Phaeocystis* and copepods). With access to these tools, chemical ecologists have an unprecedented opportunity to learn more about the biochemistry and ecological functioning of pelagic systems more quickly and comprehensively than ever. Transcriptome analyses, for example, have already been used to monitor the stress response of the diatom *Phaeodactylum tricornutum* [117] showing that the gene responsible for NO generation, PtNOA, is up-regulated in response to decadienal and that overexpressing cell lines are hypersensitive to sublethal levels of this aldehyde. This is manifested by altered expression of superoxide dismutase and metacaspases, key components of stress and death pathways. Transgenic approaches for manipulating genes in key signaling and stress-related pathways in diatoms may provide the opportunity to gain further insights into the role of PUAs in controlling algal population dynamics and other trophic-level interactions.

The genome sequence for *Pseudo-nitzschia multiseries*, which will soon be available, should provide more insights into domoic acid biosynthesis, allowing researchers to profile production capabilities across different species, to better characterize their responses to environmental triggers, and to examine the molecular interactions between toxin-producing cells and bacteria. In addition, molecular toolboxes allowing genetic transformation of phytoplankton species, protein tagging or gene abundance monitoring are in development (e.g., [118]). These techniques will bring new tools for the ecological investigation of the roles of certain pathways utilizing deletion experiments and subsequent bioassays.

Given the massive genome size of marine dinoflagellates, whole genome sequencing remains impractical. A functional genomics approach, involving generation of a cDNA library and sequencing of expressed sequence tags (ESTs), has proven useful in the search for putative biosynthetic genes for secondary metabolites, stress response genes and those involved in growth regulation leading to bloom formation [119,120]. Particularly when coupled with DNA microarrays, molecular approaches have the potential to reveal novel pathways that are up- or down-regulated during certain stress situations and might reveal the biochemical and chemical basis for some observed species-interactions that cannot be explained to date. One detailed study of intra-population diversity within the toxigenic dinoflagellate *Alexandrium tamarense* [121] revealed that the abundant genetic diversity, determined by two independent molecular markers, was not directly correlated with the phenotypic variation in toxin spectrum and intracellular concentration.

Another powerful post-genomics tool, advanced metabolite profiling, also termed “metabolomics”, allows mapping of the chemical diversity and dynamic range of bioactive secondary metabolites of potential importance in ecological interactions. Thus, the response of phytoplankton cultures or communities to competing organisms, predators and viruses can be monitored with less bias towards a certain compound class than classical studies targeting certain compounds. Statistical evaluation of the data provides insight into the metabolic changes within the cells as well as into the released metabolites that might represent the chemical language of the cells [122]. It is likely that knowledge of the complex patterns of chemicals released by microalgae and other plankton species will result in a paradigm shift revealing new mechanisms for processes such as: community function, food location, or complex defensive and allelopathic interactions. The merging of metabolomics and metagenomics has the potential to be a particularly powerful combination in shedding light on planktonic and microbial interactions in marine ecosystems [123].

Another important tool in plankton chemical ecology studies will be the adoption of model organisms that will provide insight into the functioning of other organisms. This approach is widely used in the biomedical field to explore potential causes and treatments for human disease and is based on the concept of conservation of biochemical pathways and genetic material over the course of evolution. Models are chosen on the basis that they are suitable for experimental manipulation, have characteristics such as a short life-cycle, are amenable for maintenance and breeding in the laboratory, and have non-specialist living requirements. In terrestrial chemical ecology, the introduction of *Arabidopsis thaliana* as the first fully sequenced plant proved to be highly successful for the elucidation of rather general plant defensive pathways (e.g., [124]). Such an approach could also be useful in the plankton and would allow for the elucidation of fundamental biochemical mechanisms such as regulatory and metabolic pathways in producer organisms as well as the identification of the mechanisms of action in target organisms. Ideally, the models selected in plankton ecological studies would also be supported by genomic and post-genomic resources, databases and infrastructure.

## 6. Conclusions and Future Directions

**Figure 2:**
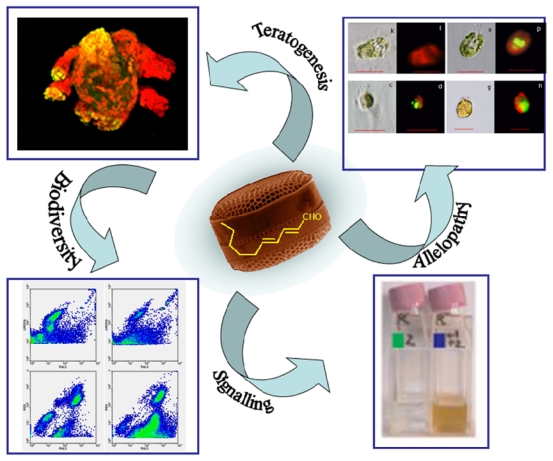
Multiple effects of diatom polyunsaturated aldehydes (PUAs): Upper left, anti-predatory (teratogenic) effect on copepods. Abnormal *Calanus helgolandicus* copepod nauplius hatched from a mother fed with the PUA-producing diatom *Skeletonema marinoi*. Yellow parts indicate TUNEL-positive apoptotic tissues (from [16]). Upper right, allelopathic (anti-growth) effect on phytoplankton species other than diatoms in culture. Clockwise from upper left: *Dunaliella tertiolecta*, *Tetraselmis suecica*, *Isochrysis galbana* and *Amphidinium carterae*, after 48 h exposure to PUA, light transmitted (left) and epifluorescence microscopy after DNA staining with SYTOX Green, (from [17]). Lower left, effect on community composition of picoplankton. Flow cytograms represent a natural seawater sample from the Adriatic Sea; left panels are controls, right panels are picophytoplankton (top) and heterotrophic bacteria (bottom), after 24 h inoculation with a mixture of octadienal and heptadienal. Lower right, signaling effect. Sublethal doses of PUA confer resistance to further doses of PUAs. Pre-treated cultures of *Phaeodactylum tricornutum*, are able to recover after release from PUA exposure compared to non-pretreated cultures (left flask, from [18]).

Future plankton studies will need to address the problem of multiple simultaneous functions of many of these secondary metabolites and derivatives of primary metabolism (referred to as “keystone molecules” or “molecules of keystone significance” [125,126]). Wink and Schimmer [127] explained the evolution of such compounds in terms of nature’s tendency “to catch as many flies with one clap as possible”. For example (Figure 2), diatom PUAs may have an anti-predatory function [16] and also act as allelopathic agents [17]. Furthermore, PUAs can affect growth of some bacterial strains [19] and possibly also have a stress signaling function [18], with potential consequences for food web structure and community composition. Thus, the same secondary metabolites may act to deter different groups of organisms by different modes of action. Wink [128] claims that multiple functions of secondary metabolites in terrestrial plants are common and do not contradict their main role for chemical defense and signaling. Furthermore, he argues that natural selection will favor those metabolites that possess multiple functions. Future ecological studies of plankton secondary metabolites will therefore have to consider such multiple functions and the multiple pathways by which metabolites mediate chemical interactions among organisms and how these in turn are mediated by biotic and abiotic environmental factors.

Another important consideration is that herbivores, competitors and pathogens do not interact with chemicals in isolation. For example, copepods that ingest PUAs simultaneously consume several other compounds that derive from the oxidation of fatty acids, as well as other secondary metabolites produced by diatoms. Likewise, diatom competitors and pathogens are exposed to a bouquet of compounds released through lysis of the cell. Given the complexity of chemical interactions a common approach in chemical ecology has been to quantify the total amount of all compounds that belong to a single class (e.g., PUAs) and to associate them to a common biological property. Several of these compounds indeed have a common activity but because there is the potential for chemical interactions and synergism between these molecules and other secondary metabolites, isolated ecological functions may not accurately reflect the overall biological activity of these compounds.

Herbivores also vary profoundly in their tolerance to secondary metabolites and future research should be directed to better understanding detoxification processes, or lack of such processes, in the plankton. Sotka and Whalen [68] termed this tolerance both a blessing and a curse. On the one hand, there is increasing evidence for the importance of key detoxification enzymes with broad substrate specificities [68]. At the same time, each herbivore species is likely to show unique features in detoxification/transport mechanisms as is the case for detoxification in insects. The complexity of planktonic food webs allows a multitude of pathways for infochemicals that need to be better understood. The role of microzooplankton, parasites and viruses, both in introducing toxins into the plankton, as well as removing them through detoxification or species-specific host-pathogen interactions is poorly known. Resulting trophic cascades may have important repercussions in mesozooplankton structure with implications for higher trophic levels.

We have only barely begun to understand the importance of chemical interactions in the plankton and their role in shaping biodiversity and ecological functioning both at a community and cellular level. An increased understanding of chemical aspects of species-specific organism interactions, including symbiotic relationships between microalgae and their bacterioflora, will help define the chemical and molecular processes that are crucial for the maintenance of biodiversity and ecosystem functionality in the plankton.

## Figures and Tables

**Table 1:** A synopsis of the effects of polyunsaturated aldehydes (PUAs) and other microalgal metabolites on predators, competitors and pathogens in the plankton.

Compounds		Producer	Target organism	Effect	Mode of action	References
PUAs	Decatrienal	*Thalassiosira rotula* and other *Thalassiosira* species	Copepods Phytoplankton Bacteria	Reduced hatching Abnormal nauplii Growth inhibition	Anti-mitotic Apoptosis	[6,7,17,19]
	Octatrienal Heptadienal	*T. rotula* *Skeletonema marinoi*	Copepods Phytoplankton	Reduced hatching Abnormal nauplii Growth inhibition	Anti-mitotic Apoptosis	[23,24]
Hydroxy- and epoxy-Fatty acids		*T. rotula* *S. marinoi* *Pseudo-nitzschia delicatissima* *Chaetoceros affinis* *C. socialis*	Copepods	Reduced hatching Teratogenic effect Growth inhibition	Anti-mitotic Apoptosis	[8,25,26]
Fatty acid hydroperoxydes		*T. rotula* *S. marinoi* *P. delicatissima* *C. affinis* *C. socialis*	Copepods	Reduced hatching Teratogenic effect Growth inhibition	Anti-mitotic Apoptosis	[8,25]
Oxoacids		*P. delicatissima*	Copepods	Reduced hatching Teratogenic effect	Anti-mitotic Apoptosis	[9,27]
DMSP: acrylate DMS		*Emiliana huxleyi*	Protistan Crustacean	Feeding deterrence Search behaviour	Unknown	[28]
Apo-fucoxanthins		*T. weissfloggi* *Phaeodactilum tricornutum*	Copepods	Feeding deterrence	Unknown	[29,30]
Saxitoxins		*Alexandrium* sp.	Copepods	Feeding deterrence	Unknown	[31]
Karlotoxin		*Karlodinium veneficum*	Copepods Cryptophytes	Feeding deterrence Prey capture	Unknown	[32]
Lytic compound (s)		*Alexandrium tamarense*	*Rhodomonas baltica*	Cell membrane lyis	Unknown	[33]
